# Differences in HIV knowledge and sexual practices of learners with intellectual disabilities and non-disabled learners in Nigeria

**DOI:** 10.7448/IAS.16.1.17331

**Published:** 2013-02-08

**Authors:** Toyin J Aderemi, Basil J Pillay, Tonya M Esterhuizen

**Affiliations:** 1 Ayahulet Consulting, Ibadan, Nigeria; 2 Department of Behavioural Medicine, Nelson R Mandela School of Medicine, University of KwaZulu-Natal, Durban, South Africa; 3 Programme of Bio & Research Ethics and Medical Law, College of Health Sciences, University of KwaZulu-Natal, Durban, South Africa

**Keywords:** HIV, intellectual disabilities, sexual practices, knowledge, adolescents, Nigeria

## Abstract

**Introduction:**

Individuals with intellectual disabilities are rarely targeted by the current human immunodeficiency virus (HIV) response, thereby reducing their access to HIV information and services. Currently, little is known about the HIV knowledge and sexual practices of young Nigerians with intellectual disabilities. Thus, this study sought to compare the HIV knowledge and sexual practices of learners with mild/moderate intellectual disabilities and non-disabled learners (NDL) in Nigeria. Findings could help in the development of HIV interventions that are accessible to Nigerian learners with intellectual impairments.

**Methods:**

This cross-sectional, comparative study utilized a survey to investigate HIV knowledge and sexual practices among learners with mild/moderate intellectual disabilities and NDL in Nigeria. Learners with mild/moderate intellectual disabilities (*n=*300) and NDL (*n=*300) within the age range of 12 to 19 years drawn from schools across Oyo State, Nigeria, completed a structured questionnaire to assess their knowledge of HIV transmission and sexual practices.

**Results:**

Significantly more learners with mild/moderate intellectual disabilities (62.2%) than NDL 48 (37.8%) reported having sexual experience (*p*=0.002). Of the sexually experienced female learners with mild/moderate intellectual disabilities, 28 (68.3%) reported history of rape compared with 9 (2.9%) of female NDL (*p*=0.053). Intellectual impairment was significantly associated with lower HIV transmission knowledge scores (*p*<0.001). Learners with mild/moderate intellectual disabilities were less likely than NDL (*p*<0.001) to have heard about HIV from most of the common sources of HIV information. In addition, when compared with non-disabled learners, learners with mild/moderate intellectual disabilities were significantly more likely to have reported inconsistent condom use with boyfriends/girlfriends (*p*<0.001), with casual sexual partners (*p*<0.001) and non-use of condom during last sexual activity (*p*<0.001).

**Conclusions:**

Findings suggest that adolescents with intellectual impairments are at higher risk of HIV infection than their non-disabled peers. This gap could be addressed through interventions that target Nigerians with intellectual impairments with accessible HIV information and services.

## Introduction

Nigeria, with HIV prevalence of 3.6% and 2.98 million persons living with HIV (PLHIV), is the worst hit country in west Africa, and second to South Africa in the number of PLHIV [1]. Heterosexual sex (80.0%) is the main mode of transmitting HIV infection in Nigeria, and contributing factors include lack of access to sexual health and HIV information and services [2]. Despite a lot of research on HIV risk factors among different populations, there is a dearth of such research focussing on persons with disabilities, particularly adolescents with intellectual impairments, in Nigeria. Consequently, little is known about the risk factors for HIV infection among adolescents with intellectual disabilities in Nigeria, and whether or not they face higher risk of HIV infection than their non-disabled peers. This study sought to investigate and compare HIV knowledge and sexual practices of learners with mild/moderate intellectual disabilities and non-disabled learners (NDL) in Nigeria.

Until recently, persons with disabilities – physical, sensory (blindness and deafness), intellectual and mental – had been overlooked in HIV response despite their equal or higher exposure to HIV [3]. They were often assumed not to be at risk of HIV infection due to erroneous beliefs that they were sexually inactive, unlikely to use drugs/alcohol and less likely to be raped than non-disabled people [4]. Groce [5] asserts that individuals with disabilities are among the world's most stigmatized, poorest and least educated citizens. Thus, they face lack of access to healthcare, poverty, social inequality and lack of human rights protection [6,7].

Persons with disabilities constitute approximately 15% of the world's population [8], making them the largest minority group globally [9]. In addition, 80% of the estimated over a billion persons with disabilities live in the developing countries [10], and over 75 to 150 million adolescents and youth with disabilities may be living in developing countries [11]. Experience of poverty, social inequality, lack of human rights protection and lack of access to healthcare [6,7] may place them at a higher risk of contracting HIV than their non-disabled peers.

Research reveals inadequate sexuality education/information for youth with disabilities at home and in schools due to communication barriers, discomfort about sexuality and disability, concerns about appropriate contents of sexuality education and fear of promoting sexual activities [12,13]. For persons with intellectual disabilities this is worsened by the social construction of their expression of sexuality as “abnormal” [13,14]. Previous research findings indicate that persons with disabilities access HIV and sexual and reproductive health (SRH) information from a range of sources but mostly from radio, television and health professionals [15–17]. However, available evidence suggests less access to HIV/SRH information and services and more risky sexual practices among persons with intellectual disabilities than other persons with disabilities [15,18]. There are documentations of inadequate HIV transmission and prevention knowledge, as well as risky sexual behaviours among persons with different impairments, with female tending towards lower HIV knowledge than male [15,16,19,20]. In addition, in South Africa, studies had shown low HIV knowledge and risky sexual exposures among adolescents with intellectual disabilities as a group [21,22]. A study also documented lower HIV knowledge among youth with intellectual impairments than non-disabled youth in Australia [23].

Although HIV prevalence studies among persons with disabilities are still sparse in Africa, a recent study in South Africa found a HIV prevalence of 12.5% among sexually abused female adolescents with mental disabilities [24]. Furthermore, a 2008 South African national study indicates a HIV prevalence of 14.1% among persons with disabilities which is close to the national prevalence of 16.9% among age group 15 to 49 [25]. Similarly, among deaf populations in Yaounde, Cameroon, the HIV prevalence was 4%, which was similar to the prevalence of 4.7% in the city [26]. And over a two-year period, 7% HIV prevalence was documented in Kenya [27]. These studies serve as evidences that Africans with disabilities are indeed at the risk of HIV infection.

Numerous studies had been undertaken to document HIV knowledge, attitudes and sexual practices of Nigerian adolescents, and the shift is now towards determining effective interventions in this group [28–32], whereas only very few are available on adolescents with disabilities. Studies on HIV knowledge and sexuality of adolescents with disability are necessary to provide baseline information for tailored sexuality and HIV education for this group of individuals.

Despite a call for research on disability and HIV by Groce [3], in Nigeria none of the prevailing studies were primarily targeted at adolescents with intellectual disabilities. Currently, there are only six published papers on HIV and disabilities in Nigeria, three of which focused specifically on the deaf population [33–35]. A comparative study by Groce [33] reported differences in levels of understanding of HIV transmission, as well as differences in access to HIV information among persons who are deaf and non-disabled persons. Similarly, Olawuyi [34], in a study documenting the causes of HIV prevalence among population of deaf persons in Nigeria, found that there was a lack of access to HIV information in his sample; only 10% had adequate knowledge of HIV transmission and most participants engaged in risky sexual behaviours. In another study Osowole [35] evaluated the effect of HIV education (delivered through peer education) on HIV knowledge, attitude and perceived susceptibility among Nigerian secondary school students who are deaf. She found that while peer education intervention was effective in improving HIV knowledge of her respondents, it was limited in changing their perceived susceptibility to HIV infection.

Another Nigerian study [36] to investigate HIV prevention needs and to identify gaps in disability-inclusive HIV response in two settlements for persons who are deaf and those who have leprosy documented a poor knowledge of HIV transmission among participants. In addition, the relevant authorities in the settlements were not committed to the provision of SRH and HIV prevention services and there was a discriminatory attitude towards HIV prevention activities in such settlements. Moreover, another comparative study of HIV knowledge and accessibility to HIV information among blind and sighted adolescents found an association between blindness and low knowledge of HIV transmission, prevention and symptoms [37]. Blind adolescents also reported limited access to HIV information than their sighted peers.

Furthermore, a study [18] explored sexual behaviours and reproductive health knowledge of in-school young persons with hearing, visual, speech, intellectual and physical disabilities in Ibadan, Nigeria (only 10 of the 103 participants had intellectual impairments). They found that 35% of their participants were sexually experienced. The study also showed that generally, inconsistent condom use was high and more than a quarter of the participants had a history of rape. A higher proportion (8 out of 10) of respondents with intellectual disabilities was sexually experienced and multiple sexual partnering was most common among participants with intellectual impairments (50% reported having more than one sexual partner). Exposure to HIV educational programmes was lowest among those with hearing and speech, speech and intellectual impairments. Overall, 70% of the sample had no knowledge of where to obtain reproductive health services, if they were in need of it.

Adolescents with intellectual impairments are often more marginalized and less knowledgeable in sexual matters than other adolescents with disabilities due to difficulties with: learning and retaining information [38], inadequate sex education [39] and inadequate information regarding the emotional and psychological aspects of intimate relationships. In addition, Olaleye *et al*. [18] suggest that in Nigeria, people with intellectual disabilities are more marginalized than other persons with disabilities in accessing HIV-related services due to their cognitive disabilities. Generally, it has been documented that parents either do not believe that they need such education or lack the skills to convey such issues in accessible formats [13]. The school therefore may be a good alternative for learning sexuality and HIV-related information for learners with intellectual disabilities.

Contrary to being taught from primary to tertiary level of education as planned [40], Nigerian schools currently teach Family Life and HIV Education at junior secondary school level by including it in the curricula of Social Studies and Basic Science [41]. Therefore, learners with mild/moderate intellectual disabilities (LMID) hardly have access to sexuality education in Nigeria because they are mostly at the primary level of education. Only a few do reach secondary schools.

It has been suggested that properly channelled sexuality and HIV education will not only increase the knowledge of persons with mildly/moderately intellectual impairments, but will also equip them with skills for modifying sexual behaviours [42]. However, such educational packages are not yet available in Nigeria. Therefore, there is need for baseline data to guide the development of tailored sexuality and HIV education for learners with intellectual disabilities in Nigerian schools. This study documents the levels of HIV knowledge and sexual practices among LMID compared with those of NDL in Nigeria.

## Methods

This comparative, cross-sectional study was undertaken in Oyo State, Nigeria. Oyo State, with a population of 5.58 million [43], is one of the six states in the south-west of Nigeria, predominantly of Yoruba ethnicity. A sample size of 257 was calculated for each group of LMID and NDL to be sufficient to analyze differences between the two groups. It was increased to 300 for each group to allow for non-response and other related challenges that may arise. The study population was made up of LMID and NDL aged 12 to 19 years in public primary and secondary schools in the six educational zones with learners with intellectual impairments.

Multi-stage sampling procedures were followed to recruit NDL from six secondary schools, whereas all eligible LMID in 12 special schools in the six educational zones were included to achieve the required sample size. In each secondary school, 50 learners were randomly selected from each arm of five classes representing junior secondary classes 1 to 3 and senior secondary classes 1 to 2 (excluding senior secondary 3 students who were writing examinations during the period). Participants were accepted into the study if they were: (1) LMID in educable or trainable classes; (2) mainstream learners without intellectual impairments; (3) learners in schools within the six educational zones that have schools for learners with disabilities; (4) aged 12 to 19 years; and (5) capable of, and provided informed consent. Intellectual impairments and adaptive behaviours were established by administering the Raven's Progressive Matrices [44,45] and Draw-A-Person Test [46] to all participants, and the Vineland's Social Maturity Scale [47] to caregivers of LMID.

A structured questionnaire was self-administered by NDL in a school hall, laboratory or classroom in the absence of teachers and other learners who did not participate in the study. Trained research assistants (two male and two female graduates aged 24 to 32 with previous research experience) read the questions along with them in groups in Yoruba language to aid understanding. The trained research assistants administered the same questionnaire individually to LMID. Pictorial cards, mostly adapted from a manual on sexuality and HIV education for young people with intellectual disabilities [48], were used as adjuncts to illustrate questions on sexuality and HIV transmission to ensure understanding of the concepts among both groups. LMID who gave inconsistent responses during interviews were dropped.

The questionnaire, adapted from a previous study [49], elicited information on demographics, sexual behaviours, sources of HIV information, HIV transmission knowledge and substance use of learners. To ensure that learners with intellectual impairment understood the questions, they were framed using simple, non-ambiguous language. Because people with intellectual disabilities are particularly prone to response biases [50,51], the approach recommended by Sigelman *et al*. [52] to avoid questions eliciting either overt or passive responses, and a careful design of questionnaires and interviews to optimize responsiveness, reliability and validity, was employed. To reduce acquiescence among LMID and to inject a conversational tone into the questions [53,54], yes/no questions ended with “or not” (e.g. “have you ever had sex, or not?”).

### Reliability and validity

To ensure reliability, the questionnaire was translated into Yoruba and then translated back into English by another Yoruba-speaking researcher before and after the pilot study. Necessary corrections were made to obtain a correct Yoruba version of the instruments. A researcher in the field of disability and HIV went through the final English version of the questionnaire to confirm face and content validity. To identify comprehension of the contents, time required to administer and other challenges the questionnaire was piloted among LMID and NDL that were similar to the study population. Also during the pilot, other terminologies of sexual concepts in use by both groups of learners; the need for flexibility in response format of LMID; and the need to always check understanding of concepts were identified and addressed in the final questionnaire.

### Ethical considerations

Written approvals were obtained for the study from the Biomedical Research Ethical Committee of the University of KwaZulu-Natal, Durban, South Africa and the HIV and AIDS Desk, Oyo State Ministry of Education, Nigeria. Based on this, the school principals/head teachers gave permission to conduct the study. Parents/guardians indicated if they did not want their wards to participate in the study after informing them about the study through parent–teachers associations and in written form through the learners. Furthermore, informed consent was obtained from all participants before they were recruited. LMID were guided by the researcher and trained research assistants through an adapted informed consent procedure (utilized simple language and assessed understanding and willingness to participate) that had been proven to be appropriate for individuals with intellectual impairments [55]. LMID were not included in the study if they demonstrated lack of understanding of what the study was about or did not agree to participate.

### Data analysis

Data analysis was done using SPSS 15.0. Bivariate analyses entailed t-tests, Pearson's correlation analysis and ANOVA for continuous variables while Pearson's Chi-square/Fisher's Exact tests were used to analyze dichotomous variables. A *p*-value<0.05 was considered as statistically significant.

## Results

### Characteristics of the overall data

The final analysis was based on completed questionnaires from 300 LMID (50%) and 300 NDL (50%). Of the LMID, 123 (41%) were female, whereas 154 (51.3%) of the NDL were female (*p*=0.011). There was a significant difference, *p*<0.001, between the mean age of LMID (M=16.3, SD=2.32) and NDL (M=15.4, SD=1.87). LMID (228, or 52.8%) were better represented than NDL in the age group 15 to 19 than age group 12 to 14. Similarly, 147 (79%) of learners in alternative living arrangements (not living with a father and mother) had intellectual disabilities compared with 39 (21%) of NDL in the same living arrangements (*p*<0.001). Yoruba was the language spoken at home by 506 (84.3%) of the learners. Most of the learners were either Christians (327, or 54.5%) or Muslims (271, or 45.2%).

Significantly more female 41 (32.3%) than male 38 (29.9%) LMID reported previous sexual intercourse (*p*=0.022). Of the learners that reported sexual intercourse in the past six months, 18 (37.5%) and 30 (62.5%) were LMID and NDL, respectively (*p* <0.001). Of the learners that reported having boyfriends/girlfriends, 69 (37.7%) were LMID compared to almost two-thirds (114, or 62.3%) that were NDL (*p*<0.001). The age range for learners’ first sexual intercourse was 7 to 19. Many (44, or 64.7%) of the learners (both LMID and NDL) reportedly had first sexual intercourse between the ages of 13 and 16 years. Twenty-nine (78.4%) of learners who reported that their first sexual partners were much older had intellectual impairments. All LMID (29, or 100%) who reported much older first sexual partners were female but an equal number (4, or 50.0%) of male and female NDL reported that their first sexual partners were much older (Table 1).

**Table 1:** Socio-behavioural characteristics of learners

		LMID *n* (%)			NDL *n* (%)		
					
	Sub-total	Female	Male	Sub-total	Female	Male	Total
Had a boyfriend/girlfriend							
No	231 (55.4)	97 (23.3)	134 (32.1)	186 (44.6)	104 (24.9)	82 (19.7)	417 (100.0)
Yes	69 (37.7)	26 (14.2)	43 (23.5)	114 (62.3)	50 (27.3)	64 (35.0)	183 (100.0)
History of sexual intercourse							
No	221 (46.7)	82 (17.3)	139 (29.4)	252 (53.3)	133 (28.1)	119 (25.2)	473 (100.0)
Yes	79 (62.2)	41 (32.3)	38 (29.9)	48 (37.8)	21 (16.5)	27 (21.3)	127 (100.0)
Age at first sexual intercourse							
Below 13	4 (30.8)	0 (0.0)	4 (30.8)	9 (69.2)	3 (23.1)	6 (46.1)	13 (100.0)
13–16	12 (27.2)	6 (13.6)	6 (13.6)	32 (72.7)	14 (31.8)	18 (40.9)	44 (100.0)
17–19	4 (36.4)	0 (0.0)	4 (36.4)	7 (63.6)	4 (36.4)	3 (27.2)	11 (100.0)
Do not remember	59 (100.0)	35 (59.3)	24 (40.7)	0 (0.0)	0 (0.0)	0 (0.0)	59 (100.0)
Age of first sexual partner							
Peer	44 (62.0)	8 (11.3)	36 (50.7)	27 (38.0)	9 (12.7)	18 (25.3)	71 (100.0)
A bit older	6 (31.6)	4 (21.1)	2 (10.5)	13 (68.4)	8 (42.1)	5 (26.3)	19 (100.0)
Much older	29 (78.4)	29 (78.4)	0 (0.0)	8 (21.6)	4 (10.8)	4 (10.8)	37 (100.0)
Last time of sexual intercourse							
Within past six months	18 (37.5)	14 (29.2)	4 (8.3)	30 (62.5)	10 (20.8)	20 (41.7)	48 (100.0)
Over six months	0 (0.0)	0 (0.0)	0 (0.0)	12 (100.0)	6 (50.0)	6 (50.0)	12 (100.0)
Over one year	0 (0.0)	0 (0.0)	0 (0.0)	1 (100.0)	1 (100.0)	0 (0.0)	1 (100.0)
Do not remember	61 (92.4)	27 (40.9)	34 (51.5)	5 (7.6)	4 (6.1)	1 (1.5)	66 (100.0)

*n*=300.

### Sources of HIV information

Participants were asked to indicate their sources of HIV information from a list of sources. Significantly less LMID than NDL heard about HIV from common sources of HIV information (Table 2). Radio and television were the main sources of HIV information for both LMID and NDL; and both groups reported similar access to HIV information through radio (*p*=0.132) and television (*p*=0.399). In addition, LMID were significantly less likely than NDL to have heard about HIV from newspapers (*p*<0.001), books (*p*<0.001) and magazines (*p*<0.001). Female and male NDL had similar access to HIV information through the various sources investigated (Table 2). But male LMID reported better access than female LMID to HIV information through parents (*p*=0.001), brothers/sisters (*p*=0.037), friends (*p*=0.004) and teachers (*p*=0.037).

**Table 2:** Sources of HIV information

		LMID *n* (%)				NDL *n* (%)			
					
Heard about HIV from	*p*#	Female	Male	Total	*p* $	Female	Male	Total	p*
Parents	0.001	8 (18.6)	35 (81.4)	43 (100.0)	0.856	67 (51.9)	62 (48.1)	129 (100.0)	<0.001
Brothers/sisters	0.037	2 (14.3)	12 (85.7)	14 (100.0)	0.231	35 (45.5)	42 (54.5)	77 (100.0)	<0.001
Other relations	1.174	6 (27.3)	16 (72.7)	22 (100.0)	0.771	37 (52.9)	33 (47.1)	70 (100.0)	<0.001
Friends	0.004	1 (6.3)	15 (93.8)	16 (100.0)	0.520	55 (53.9)	47 (46.1)	102 (100.0)	<0.001
Radio	0.213	77 (38.5)	123 (61.5)	200 (100.0)	0.164	106 (48.8)	111 (51.2)	217 (100.0)	0.132
Television	0.081	72 (37.3)	121 (62.7)	193 (100.0)	0.469	97 (53.0)	86 (47.0)	183 (100.0)	0.399
Church/mosque	0.093	0 (0.0)	4 (100.0)	4 (100.0)	0.913	34 (50.7)	33 (49.3)	67 (100.0)	<0.001
Newspapers	0.237	0 (0.0)	2 (100.0)	2 (100.0)	0.243	74 (48.1)	80 (51.9)	154 (100.0)	<0.001
Books	0.237	0 (0.0)	2 (100.0)	2 (100.0)	0.587	40 (48.8)	42 (51.2)	82 (100.0)	<0.001
Magazines	0.237	0 (0.0)	2 (100.0)	2 (100.0)	0.642	53 (49.5)	54 (50.5)	107 (100.0)	<0.001
Hospitals/clinics	0.700	2 (33.3)	4 (66.7)	6 (100.0)	0.102	81 (56.3)	63 (43.8)	144 (100.0)	<0.001
Teachers	0.037	2 (14.3)	12 (85.7)	14 (100.0)	0.125	79 (56.0)	62 (44.0)	141 (100.0)	<0.001

# *p*-value: significance level within LMID

$ *p*-value: significance level within NDL

* *p*-value: significance level between LMID and NDL.

*n*=300.

### HIV transmission knowledge of learners

HIV transmission knowledge of participants was assessed with nine items eliciting “yes/no” responses: knowledge of sexual routes of HIV transmission (vaginal sex, anal sex, kissing), knowledge of mother-to-child HIV transmission (during pregnancy, delivery or breastfeeding) and knowledge of other routes of HIV transmission (sharing of cup, toilet, and blood contact). The scores were derived by computing the percentage of right responses given by each learner out of the nine items that measured learners’ HIV transmission knowledge. The HIV transmission knowledge scores for all learners ranged between 0% and 100.0%, with a mean score of 61.6%. Bivariate analysis showed that having intellectual impairment was significantly associated with lower HIV transmission knowledge scores (*p*<0.001). Male LMID (M=59.9, SD=24.2) were significantly more knowledgeable about HIV transmission than female LMID (M=48.4, SD=24.9), with *p*=0.009. Learners with intellectual impairments were also significantly more likely than their non-disabled peers to believe that HIV could be transmitted through kissing, sharing a toilet or cup (*p*=0.009). Table 3 shows that there were no significant differences between learners’ religious beliefs (*p*=0.112), gender (*p*=0.368), school location, *(p*=0.066), age (*r*=−0.073; *p*=0.075) and their HIV transmission knowledge scores.

**Table 3:** HIV transmission knowledge scores of learners

	HIV transmission knowledge scores	
		
	*M* (*SD*)	*p*
Groups of learners^a^		
LMID	52.85 (24.73)	
NDL	70.44 (17.42)	<0.001
Sex^a^		
Female	60.73 (24.01)	
Male	62.44 (22.33)	0.368
School location^a^		
Urban	60.44 (23.42)	
Less urban	64.13 (22.35)	0.066
Religion^b^		
Christianity	60.00 (24.15)	
Islam	63.51 (21.65)	
Other	77.78 (31.42)	0.112
Age (years)^c^	*r* =−0.073	0.075

a *t*-test

b ANOVA

c Pearson's correlation.

### Sexual experience

Learners were asked to indicate whether or not they had engaged in sexual intercourse before. Bivariate analysis indicated significant associations between sexual experience (history of sexual intercourse) and intellectual impairment (*p*=0.002); the age group of 15 to 19 (*p*<0.001); and having a boyfriend/girlfriend (*p*<0.001). There were no significant differences between sexual experience and religious beliefs (*p*=0.502); languages spoken at home (*p*=0.556); living arrangements (*p*=0.233); HIV transmission knowledge scores (*p*=0.141); use of cigarettes (*p*=0.102), marijuana (*p*=0.612) and other hard drugs (*p*=0.234).

### Sexual practices

To assess learners’ sexual practices, they responded with always/often/sometimes/rarely to statements on condom use with boyfriends/girlfriends, condom use with someone new to them, and sexual intercourse with someone else when in a relationship. Condom use at last sexual intercourse was assessed through a yes/no response.

### Condom use

Table 4 shows that all sexually experienced LMID 79 (100%) reported inconsistent condom use with their boyfriends/girlfriends while only 26 (45.2%) of NDL reported the same (*p*<0.001). Similarly, more LMID 76 (96.2%) than NDL 28 (58.3%) reportedly practised inconsistent condom use with casual sexual partners (*p*<0.001). In addition, reported condom use at last sexual intercourse was significantly lower (*p*<0.001) among LMID 4 (5.1%) compared with NDL 19 (39.6%). Female NDL 16 (33.3%) were more likely than their male peers 10 (20.8%) to report inconsistent condom use with boyfriends/girlfriends (*p*=0.007).

**Table 4:** Sexual practices of learners

		LMID *n*_*1*_ (%)				NDL *n*_*2*_ (%)			
					
	*p* #	Female	Male	Total	*p* $	Female	Male	Total	p*
Condom use with boyfriends/girlfriends									
Always/often		0 (0.0)	0 (0.0)	0 (0.0)		5 (10.4)	17 (35.4)	22 (45.8)	
Sometimes/rarely	–	41 (51.9)	38 (48.1)	79 (100.0)	0.007	16 (33.3)	10 (20.8)	26 (54.2)	<0.001
Condom use with casual sexual partners									
Always/often		2 (2.5)	1 (1.3)	3 (3.8)		7 (14.6)	13 (27.1)	20 (41.7)	
Sometimes/rarely	0.602*	39 (49.4)	37 (46.8)	76 (96.2)	0.302	14 (29.2)	14 (29.2)	28 (58.3)	<0.001
Condom use at last sexual intercourse									
No		24 (30.3)	38 (48.1)	62 (78.4)		14 (29.2)	14 (29.2)	28 (58.4)	
Yes		4 (5.1)	0 (0.0)	4 (5.1)		7 (14.5)	12 (25.0)	19 (39.5)	
Do not remember	<0.001*	13 (16.5)	0 (0.0)	13 (16.5)	0.451*	0 (0.0)	1 (2.1)	1 (2.1)	<0.001
Multiple sexual partners									
Always/often		10 (12.7)	6 (7.6)	16 (20.3)		6 (12.5)	15 (31.3)	21 (43.8)	
Sometimes/rarely	0.342	31 (39.2)	32 (40.5)	63 (79.7)	0.062	15 (31.2)	12 (25.0)	27 (56.2)	0.005
No. of sexual partners in the last six months									
1		23 (43.4)	11 (20.8)	34 (64.2)		7 (21.9)	7 (21.9)	14 (43.8)	
>1	0.070	8 (15.1)	11 (20.7)	19 (35.8)	0.198	5 (15.6)	13 (40.6)	18 (56.2)	0.066

# *p*-value: significance level within LMID

$ *p*-value: significance level within NDL

* *p*-value: significance level between LMID and NDL.

* Pearson Chi-square test was invalid because more than 20% of cells have expected counts less than 5.

*n*_*1*_=79; *n*_*2*_=48.

### Sexual partners

NDL were significantly more likely to have multiple sexual partners than LMID (*p*=0.005). However, 16 (20.3%) of LMID reported that they always or often had multiple sexual partners (Table 4). There was no significant difference between LMID and NDL regarding the number of sexual partners in the past six months, although the trend was that more of the sexually experienced NDL than LMID had more than one sexual partner in the past six months.

### History of rape

Participants were asked to indicate whether or not they had been forced to have sexual intercourse against their will before. Twenty-eight (68.3%) of the 41 sexually experienced female LMID reported history of rape compared with 9 (2.9%) of 21 sexually experienced female NDL (*p*=0.053). However, among the boys, there was no significant difference between LMID and NDL on reports of history of rape (*p*=0.198).

### Substance use

If they use substance, learners indicated their frequency of substance use by responding with occasionally/daily/during the week and weekends/on weekends only. Twenty-three (72.4%), nine (75.0%) and two (33.3%) of the learners that reported occasional use of alcohol, cigarettes and marijuana, respectively, had intellectual impairments compared with eight (25.8%), three (25.0%) and four (66.7%) of NDL who reported the same. Furthermore, there were no significant differences between the number of sexual partners in the past six months and the use of cigarettes (*p*=0.133), alcohol (*p*=0.156), marijuana (*p*=0.187) and other hard drugs (*p*=0.435). Similarly, no significant association existed between condom use during last sexual activity and the use of cigarettes (*p*=0.655), alcohol (*p*=0.180), marijuana (*p*=1.000), and other hard drugs *(p*=1.000).

## Discussion

The findings of this study indicate that a higher proportion of LMID than NDL reported sexual experience. Learners with intellectual impairments were more likely than NDL to report risky sexual exposures such as inconsistent condom use with boyfriends/girlfriends and with casual sexual partners, as well as rape. NDL were more likely to report multiple sexual partners than LMID, and female NDL were also more likely than male NDL to practise inconsistent condom use with boyfriends. Learners with intellectual impairments had less access to sources of HIV information and demonstrated lower HIV transmission knowledge than their non-disabled peers. Gender differences were evident among LMID with regards to access to HIV information from significant others, HIV transmission knowledge, incidence of rape and having much older first sexual partners. This calls for gender-sensitive sexuality and HIV prevention education among LMID.

One of the main limitations of the study was the difference in sampling procedures utilized for LMID and NDL, which made it difficult to control for design effect in the analysis. While the design effect applied to NDL in this study, it did not apply to LMID because the sampling was not multi-stage due to small number of LMID in schools. Therefore, due to loss of efficiency it is possible that some of the differences within NDL group that appeared insignificant might have been significant. However, the analysis showed that the within and between groups differences were consistent for most of the constructs investigated. Another limitation of the study was that it relied on recall of past sexual encounters which has implication for recall bias. Recall bias may be worse among participants with intellectual impairments due to memory deficits which is common among this group [56].

Although a higher proportion of LMID reported sexual experience compared with NDL, many of the learners from both groups initiated sexual intercourse between ages 13 and 16. In addition, more NDL than LMID reported having boyfriends/girlfriends which may explain the reason for higher reports of recent sexual intercourse among NDL, while they seldom use condom with such sexual partners. However, the findings are limited by the high proportions of “do not remember” responses by LMID which could be due to memory deficit characteristic of individuals with intellectual impairments or over-reporting of sexual activities by LMID. But the findings indicate that both groups of learners may benefit from age-specific sexuality and HIV education to help the very young adolescents with and without intellectual impairments delay sexual debut and to equip the older and sexually active ones with skills to practise safer sex. Moreover, the fact that a higher proportion of female adolescents with intellectual disabilities reported having much older first sexual partners than their non-disabled peers calls for attention. A similar finding was reported by Gilbert [22] in South Africa. Having sex with older men, in particular, has been shown to carry a higher risk of HIV infection [57]. This kind of sexual relationship exposes girls with intellectual disabilities to unbalanced power dynamics and abusive relationships, in addition to risk of exposure to HIV infection.

Furthermore, girls with intellectual impairments in this study experienced more exposures to rape than non-disabled girls. This may account for higher representation of girls among LMID that reported sexual experience in the study. Although there are no previous studies in literature that compare the rate of rape among girls with and without intellectual disabilities, existing evidence confirms that girls with intellectual disabilities are easy targets for sexual violence because perpetrators are aware that due to the cognitive impairments of their victims, these individuals find it difficult to recognize their perpetrators, avoid violent situations, report such abuse and/or receive justice from the courts of law [58–60]. Thus, interventions to reduce the risk of HIV infection among individuals with intellectual disabilities should have gender perspectives and should include skills to assess, avoid and report sexual violence.

This study further confirms that although most learners with intellectual disabilities heard about HIV on radio and television, the effectiveness of such media in educating this group of adolescents is doubtful. Participants with intellectual impairments in this study demonstrated lower HIV transmission knowledge compared with NDL despite reported high exposure to HIV information through radio and television. Similar finding was documented in Australia [23]. In Africa, low HIV knowledge had also been reported among learners with intellectual disabilities although those studies were not comparative in nature [18,21].

The findings of the study suggest that radio and television may be good in raising awareness on the topic among both NDL and LMID. However, radio and television may be limited in imparting HIV knowledge to LMID because they rarely present HIV messages in formats that are accessible to persons with intellectual impairments. When properly tailored, individuals with intellectual disabilities can benefit from HIV education that specifically targets them [61]. Another proof that HIV education in the appropriate format can actually increase HIV knowledge of learners with intellectual disabilities can be drawn from the study of Gilbert [22] which showed that participants of the study had a high level of HIV knowledge because they had been previously exposed to tailored HIV education in the school setting. Therefore, sexuality and HIV information on radio and television should incorporate elements that make it accessible to individuals with intellectual impairments such as simple language and self-explanatory audio-visuals.

Even among NDL, it has been suggested that such media are more effective in improving HIV knowledge when combined with other reliable sources of HIV information such as parents, teachers and health clinics [28]. On the contrary, learners with mild or moderate intellectual impairments in this study had considerably lower access to HIV information from parents, hospitals, newspapers and teachers compared with their peers without disabilities. This is in line with reports of other studies that suggest that persons with disabilities have inadequate exposures to HIV information at homes and in schools because parents and teachers often lack expertise to give such information and/or due to the fear that it will make them promiscuous [13,14]. Here again, there is support for collaborations between parents, teachers, disability experts and health care providers in provision of HIV prevention and sexuality education to adolescents with intellectual disabilities.

Although studies on substance use among youth with intellectual impairments have to date yielded conflicting results [62–64], the finding that LMID in the current study used cigarette and alcohol occasionally more than non-disabled youth had also been documented elsewhere [65]. This should not be taken lightly given that substance use could worsen short attention spans, distortion of abstract cognitive concepts and overly compliant dispositions among persons with intellectual impairments. Further, a major consequence of substance use is the increased risk of HIV infection.

## Conclusions

The findings of this study suggest that adolescents with intellectual disabilities may be at a higher risk of HIV infection than their non-disabled peers. However, their access to HIV information was lower than that available to their non-disabled peers despite their risk of infection. There is therefore an urgent need for culturally sensitive interventions; in formats that are specifically appropriate and accessible to individuals with cognitive impairments. To achieve this, strong collaborations between stakeholders such as parents, teachers, disability experts and other HIV prevention service providers are crucial.

## Abbreviations

PLHIV, persons living with HIV; SRH, sexual and reproductive health; LMID, learners with mild/moderate intellectual disabilities; NDL, non-disabled learners.
